# Quercitrin for periodontal regeneration: effects on human gingival fibroblasts and mesenchymal stem cells

**DOI:** 10.1038/srep16593

**Published:** 2015-11-12

**Authors:** Manuel Gómez-Florit, Marta Monjo, Joana M. Ramis

**Affiliations:** 1 Group of Cell Therapy and Tissue Engineering, Research Institute on Health Sciences (IUNICS). University of Balearic Islands, Palma de Mallorca, Spain; 2 Instituto de Investigación Sanitaria de Palma, 07010 Palma, España

## Abstract

Periodontal disease (PD) is the result of an infection and chronic inflammation of the gingiva that may lead to its destruction and, in severe cases, alveolar bone and tooth loss. The ultimate goal of periodontal treatment is to achieve periodontal soft and hard tissues regeneration. We previously selected quercitrin, a catechol-containing flavonoid, as a potential agent for periodontal applications. In this study, we tested the ability of quercitrin to alter biomarker production involved in periodontal regeneration on primary human gingival fibroblasts (hGF) and primary human mesenchymal stem cells (hMSC) cultured under basal and inflammatory conditions. To mimic PD inflammatory status, interleukin-1 beta (IL-1β) was used. The expression of different genes related to inflammation and extracellular matrix were evaluated and prostaglandin E2 (PGE2) production was quantified in hGFs; alkaline phosphatase (ALP) activity and calcium content were analysed in hMSCs. Quercitrin decreased the release of the inflammatory mediator PGE2 and partially re-established the impaired collagen metabolism induced by IL-1β treatment in hGFs. Quercitrin also increased ALP activity and mineralization in hMSCs, thus, it increased hMSCs differentiation towards the osteoblastic lineage. These findings suggest quercitrin as a novel bioactive molecule with application to enhance both soft and hard tissue regeneration of the periodontium.

Periodontal disease is the result from an infection and chronic inflammation of tooth supporting tissues. Gingival inflammation or gingivitis is the first manifestation, which may lead to soft tissue destruction. In some subjects, this progresses to periodontitis, which is defined by alveolar bone and in severe cases tooth loss^1^. Current strategies for periodontal disease treatment are generally successful in eliminating active disease and some of them have achieved a certain degree of regeneration^2^. To eliminate the active disease, mechanical and antimicrobial approaches^3^ are used although the effects of local antimicrobial therapy are modest and mostly temporary, while the use of systemic antibiotics can induce the development of bacterial resistance^4^. Furthermore, these strategies alone are insufficient since periodontal disease is the result of destructive inflammation; thus, if successful, treatment frequently results in a process of gingival fibrosis and limited bone remodelling, rather than in true regeneration of the periodontal tissues^5^. In fact, there is considerable data of successful results in periodontal regeneration using guided tissue regeneration, enamel matrix derivative and platelet-derived growth factor, although the outcomes of such modalities are not always predictable^6^,^7^.

The ideal periodontal treatment should damp down the inflammatory response to either decrease the excessive production of proinflammatory mediators, destructive enzymes and free radicals and stimulate tissue regeneration, allowing for the restoration of soft tissue attachment and bone formation^8^. Recent strategies for periodontal regeneration include the use of graft materials, barrier membranes, gene therapy or growth factors through topical delivery. However, gene therapy may have undesired host immune reactions or potential tumorigenesis and growth factors are unstable and have short half-life^2^. Hence, there is an urgent need for finding bioactive molecules with both soft and hard tissue healing activities for periodontal tissue regeneration applications.

Natural phenolic compounds, such as flavonoids, are ubiquitous, abundant and offer a range of properties and an array of functions such as antioxidant^9^, anti-inflammatory^10^ and antimicrobial capacity^11^,^12^, among others. Actually, in two independent previous screenings we searched for candidate molecules for soft^13^ and hard^14^ tissue applications. From both studies, quercitrin was selected as the one showing better performance. Quercitrin decreased extracellular matrix (ECM) degradation, reduced oxidative stress levels, promoted scarless wound healing in human gingival fibroblasts and showed antibacterial properties^13^. Furthermore, it also stimulated the differentiation of mouse pre-osteoblastic cells and inhibited osteoclastogenesis from mouse monocytes, which could prevent bone resorption^14^. Moreover, as a catechol-containing flavonoid, quercitrin could help to control the inflammatory response in periodontal disease progression, since molecules with catechol moieties have shown potent anti-inflammatory effects^15^,^16^,^17^.

In the present study, we hypothesized that quercitrin could create a microenvironment suitable for periodontal regeneration due to its positive effects on soft and hard tissue cells. Thus, we set an inflammatory environment, mimicking periodontal disease, and we investigated the effects of quercitrin on primary human gingival fibroblasts and primary human mesenchymal stem cells.

## Methods

### Cell culture

Three different donors of primary human gingival fibroblasts (hGF; Provitro GmbH, Berlin, Germany) were used (range 19–47 years; male:female ratio 2:1). Provitro assured that cells were obtained ethically and legally and that all donors provided written informed consent. Cells were routinely cultured at 37˚C/5% CO_2_, and maintained in fibroblast growth medium (FGM) supplemented with 10% foetal calf serum (Provitro GmbH), amphotericin (50 ng/ml) and gentamicin (50 μg/ml).Experiments were performed with hGF between passages 7 and 8 after isolation. Three replicate wells for each donor were seeded in 96-well plates at a density of 7.0 × 10^3^ cells per well. Media was supplemented with ascorbic acid (100 μM; Sigma-Aldrich, St. Louis, MO, USA). Experiments were run in duplicate (n = 18).

Two different donors of human bone marrow-mesenchymal stem cells (hMSCs; Stemcell Technologies, Grenoble, France) were used (20 years; male:female ratio 0:2). Stemcell Technologies assured that cells were obtained ethically and legally and that all donors provided written informed consent. Cells were routinely cultured at 37 °C/5% CO_2_, and maintained in low glucose DMEM GlutaMAX (Life Technologies, Carlsbad, CA, USA) supplemented with 10% stem cell-tested foetal bovine serum (Biosera, Boussens, France), penicillin (100 U/ml) and streptomycin (100 μg/ml). Experiments were performed with hMSCs between passages 5 and 7 after isolation. Six replicate wells for each donor were seeded in 48-well plates at a density of 8.5 × 10^3^ cells per well and grown for 2 days prior to media supplementation without (control) or with additives. Experiments were run in duplicate (n = 12).

### Establishment of an inflammatory stimulus

At confluence (3 days after seeding), hGF were treated for 1 day with 0.1, 1 and 10 ng/ml of interleukin-1 beta (IL-1β; R&D systems, Abingdon, UK) diluted in complete FGM. Then cells were harvested for gene expression analysis (interleukin-6 (IL6), interleukin-8 (IL8) and matrix metalloproteinase-1 (MMP1)). After analysis, 1 ng/ml IL-1β was selected for further studies.

### Effect of quercitrin on hGF

Quercitrin (PubChem CID: 5280459; Sigma-Aldrich) was dissolved in absolute ethanol and kept at −20 °C. Stock solutions were further dissolved in culture medium, prior to use. Final concentration of ethanol (1%) was included as vehicle control group in all the experiments.

**Figure 1:**
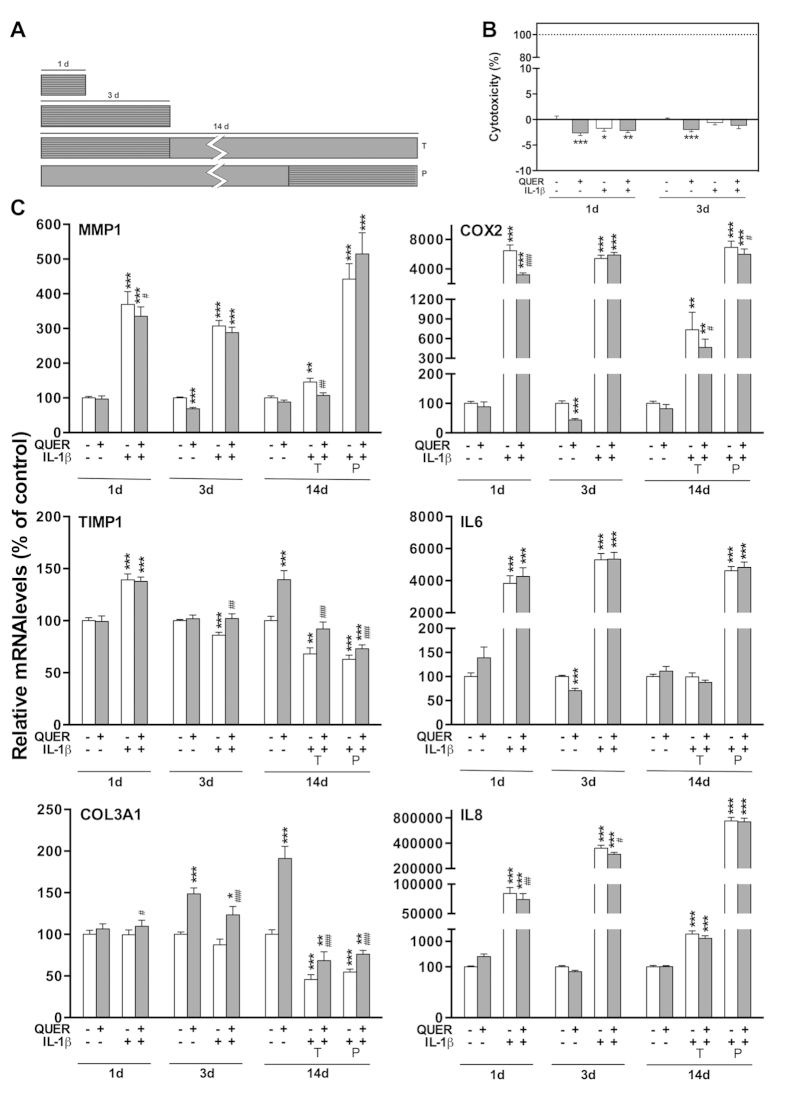
Effect of quercitrin on hGF stimulated with IL-1β. (**A**) Experimental design: the effect of quercitrin (QUER) treatment on hGF was evaluated in four different inflammatory scenarios (stripped pattern); treatment for 1 day with interleukin-1 beta (IL-1β); treatment for 3 days with IL-1β; treatment with IL-1β during the first 3 days and culturing the cells for 14 days (therapeutic approach; T); and culturing the cells for 14 days plus IL-1β treatment during the last 3 days (preventive approach; P). (**B**) Cytotoxicity measured after 1 and 3 days of treatment. Dotted line represents high control (100% cytotoxicity). (**C**) Gene expression results: cells were treated with quercitrin (grey bars) or without (white bars), following the experimental design. Data were normalized to reference genes, expressed as percentage of control (vehicle), which was set to 100%. Values represent the mean ± SEM of two independent experiments. One, two and three symbols represent a significant difference between two groups with P ≤ 0.05, P < 0.01 and P < 0.001, respectively: (*) treatment versus control (vehicle); (#) quercitrin *versus* vehicle in the presence of IL-1β for each time point.

A dose of 200 μM quercitrin was used. This dose was selected from previous studies in which different doses of quercitrin (1–500 μM) were evaluated^13^,^14^. At confluence (3 days after seeding), hGF were treated for short (1 and 3 days) and long term (14 days) with quercitrin diluted in complete FGM. Quercitrin was added at every media change (3 times per week). The inflammatory stimulus was created by the addition of IL-1β (1 ng/ml). Four different scenarios were set (Fig. 1A): Treatment for 1 day with IL-1β and quercitrin; treatment for 3 days with IL-1β and quercitrin; treatment with quercitrin for 14 days plus IL-1β during the first 3 days (therapeutic approach); and treatment with quercitrin for 14 days plus IL-1β during the last 3 days (preventive approach).

### Effect of quercitrin on hMSCs

Cells were grown under six different conditions: basal media (control); basal media with 200 μM quercitrin (QUER); osteogenic media (OS) containing ascorbic acid (50 μg/ml), dexamethasone (10 nM) and β-glycerol phosphate (10 mM); osteogenic media with 200 μM quercitrin (OS + QUER); osteogenic media supplemented with IL-1β (OS + IL-1β); and osteogenic media supplemented with IL-1β and quercitrin (OS + IL-1β + QUER). Supplements were added at every media change (twice per week). Cells were harvested after 19 days for analysis.

### Cytotoxicity and cell viability assays

The presence of lactate dehydrogenase (LDH) in culture media was used as an index of cell death. LDH activity was determined following the manufacturer’s instructions (Cytotoxicity Detection kit, Roche Diagnostics, Mannheim, Germany). Results were presented relative to the LDH activity in the medium of cells treated with the vehicle control (low control, 0% of cell death) and of cells treated with 1% Triton X-100 (high control, 100% cell death). Cytotoxicity percentage was calculated using the following equation: Cytotoxicity (%) = (exp.value − low control)/(high control − low control) × 100.

Cell viability was measured after 19 days of hMSC culture using PrestoBlue (Life Technologies, Carlsbad, CA, USA) following manufacturer’s protocol. Absorbance data was normalised to the vehicle control group (100% viability).

### RNA isolation and real-time RT-PCR analysis

Total RNA was isolated using Tripure (Roche Diagnostics, Mannheim, Germany), according to the manufacturer’s protocol. Total RNA was quantified at 260 nm using a nanodrop spectrophotometer (NanoDrop Technologies, Wilmington, DE, USA). The same amount of RNA (0.2 μg) was reverse transcribed to cDNA at 42 °C for 60 min, according to the protocol of the supplier (High Capacity RNA-to-cDNA kit, Applied Biosystems, Foster City, CA, USA). Aliquots of each cDNA were frozen (−20 °C) until the PCR reactions were carried out.

Real-time PCR was performed for two reference genes, glyceraldehyde-3-phosphate dehydrogenase and beta-actin, and target genes (Table 1). Real-time PCR was performed in a thermocycler (Lightcycler 480, Roche Diagnostics) using SYBR green detection. Each reaction contained 7 μl of master mix (Lightcycler 480 SYBR Green I Master, Roche Diagnostics), 0.5 μM of each, the sense and the antisense specific primers and 3 μl of the cDNA dilution in a final volume of 10 μl. The amplification program consisted of a pre-incubation step for denaturation of the template cDNA (5 min 95 °C), followed by 45 cycles consisting of a denaturation step (10 s 95 °C), an annealing step (10 s 60 °C) and an extension step (10 s 72 °C). After each cycle, fluorescence was measured at 72 °C. A negative control without cDNA template was run in each assay. All samples were normalized by the geometric mean of the expression levels of reference genes and fold changes were related to the control groups using the following equation: ratio = E_target_^ΔCp target (mean control − sample)^/E_reference_^ΔCp reference (mean control − sample)^, where Cp is the is the crossing point of the reaction amplification curve and E the effciency from the given slopes using serial dilutions, as determined by the software (Lightcycler 480 software, Roche Diagnostics). Stability of reference genes was calculated using a statistical tool (BestKeeper software, Technical University of Munich, Weihenstephan, Germany).

### Enzyme-linked immunosorbent assays (ELISA)

Commercially available ELISA kits were run to quantify interleukin-8 (eBioscience, San Diego, CA, USA), and prostaglandin E2 (PGE2; Thermo Scientific, Rockford, IL, USA) from cell culture media according to supplier instructions.

### Alkaline phosphatase (ALP) activity and calcium quantification

Cells were washed with PBS and lysated with 0.1% Triton X-100. ALP activity was quantified by measuring the cleavage of p-Nitrophenyl Phosphate (pNPP; Sigma-Aldrich) in a soluble yellow end product that absorbs at 405 nm. Twenty-five microliters of sample were incubated with 100 μl of pNPP. In parallel to the samples, a standard curve with calf intestinal ALP (Promega, Madison, WI, USA) was constructed. On the other hand, 200 μl of sample were incubated with HCl (1 N) overnight, followed by centrifugation at 500 × g for 2 min for the subsequent determination of Ca^2+^ content in the supernatant by inductively coupled plasma atomic emission spectrometer (Optima 5300 DV; PerkinElmer, Waltham, MA, USA). Data were compared with CaCl_2_ standards included in the assay.

### Statistical analysis

All data are presented as mean values ± standard error of the mean (SEM). The Kolmogorov-Smirnov test was done to assume parametric or non-parametric distributions. Differences between groups were assessed by paired t-test or Wilcoxon test, depending on data distribution. A specific computer program (SPSS version 17.0, Chicago, IL, USA) was used. Results were considered statistically significant at P ≤ 0.05. One, two and three symbols represent a significant difference between two groups with P ≤ 0.05, P < 0.01 and P < 0.001, respectively.

## Results and Discussion

### IL-1β induces the expression of inflammatory markers on hGFs

Here, the inflammatory mediator IL-1β was used to mimic *in vitro* the inflammatory process in periodontal disease progression. IL-1β is broadly used to induce experimental inflammation and to enhance the proinflammatory response^18^,^19^,^20^, imitating the inflammatory pathways activated in response to oral pathogens^21^. The concentrations used here are in the range with the IL-1β levels usually found in patients with periodontitis^22^. The addition of IL-1β for 1 day significantly stimulated the expression of interleukin-6 (IL6), interleukin-8 (IL8) and matrix metalloproteinase-1 (MMP1), reaching a plateau at 1 ng/mL IL-1β (Table 2). These cytokines are key inflammatory mediators in the progression of periodontal disease^21^ and the results are consistent with previous reports in the literature^20^,^23^. Moreover, hGF viability after 1 and 3 days of stimulation with IL-1β remained similar to the controls (Fig. 1B). Based on this data, the dose of 1 ng/mL IL-1β was selected to simulate a periodontitis condition although it should keep in mind that the only addition of IL-1β does not reflect the entire chronic inflammatory process in periodontal disease *in vivo*, and can only help to investigate *in vitro* the effects of quercitrin.

### Quercitrin decreases the deleterious inflammatory effects on hGFs

**Figure 2:**
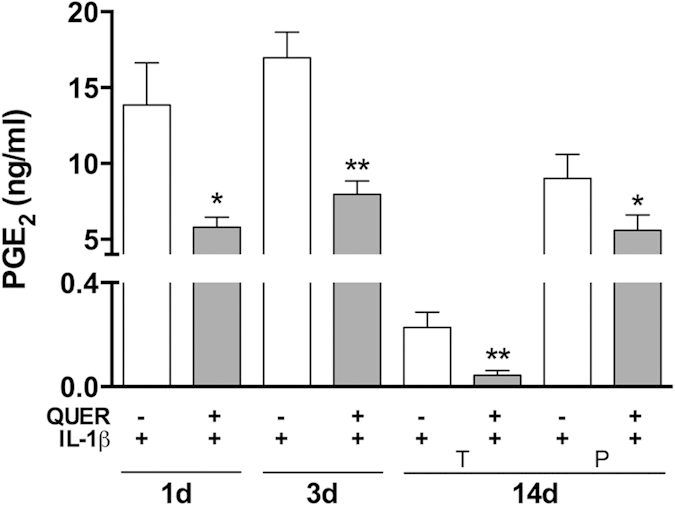
Quercitrin decreased PGE2 release on hGF stimulated with IL-1β. Cells were treated with quercitrin (QUER; grey bars) or without (white bars) in the presence of interleukin-1 beta (IL-1β). Four different scenarios were set: treatment for 1 day with IL-1β and quercitrin; treatment for 3 days with IL-1β and quercitrin; treatment with quercitrin for 14 days plus IL-1β during the first 3 days (therapeutic approach; T); and treatment with quercitrin for 14 days plus IL-1β during the last 3 days (preventive approach; P). Values represent the mean ± SEM of two independent experiments. One, two and three symbols represent a significant difference between two groups with P ≤ 0.05, P < 0.01 and P < 0.001, respectively: (*) quercitrin *versus* vehicle in the presence of IL-1β for each time point.

Modifying the local environment to reduce inflammation is one requirement to achieve complete periodontal regeneration^24^. Inducible cyclooxigenase-2 (COX2) and PGE2 production are highly related to periodontal disease^25^. Here, quercitrin treatment was not toxic (Fig. 1B) and effectively inhibited COX2 expression (Fig. 1C) and its functional product PGE2 (Fig. 2) in hGF under an inflammatory stimulus, in agreement with previous observations using different flavonoids^26^. Moreover, previous studies demonstrated that quercitrin also down-regulates inducible nitric oxide synthase, an enzyme highly expressed by inflammatory stimuli that produces the inflammatory mediator NO^27^,^28^.

The gene expression of different inflammatory markers after quercitrin treatment was also studied (Fig. 1C). Unexpectedly, the upregulation of IL6 and IL8 induced by IL-1β was not reversed by quercitrin treatmentbut for IL8 mRNA levels at short-term (days 1 and 3) although IL8 protein levels remained unchanged among groups (data not shown), contrary to what was previously proved in lipopolysaccharide-stimulated macrophages and *in vivo*^27^,^29^. This finding might be due to the stimulation with IL-1β that activates the complex NF-κB signalling pathway and positive and negative feedback mechanisms that regulate cytokines expression^30^,^31^, thus, masking quercitrin anti-inflammatory effect. Accumulating evidence suggests that the effects of phenolic compounds are mediated by interactions with signalling pathways^32^,^33^. In particular, quercitrin has been shown to have an inhibitory effect on activator protein-1 and NF-κB pathways, which have central roles regulating cell differentiation and inflammation, among other downstream targets^28^,^34^.

During the progress of gingivitis, the inflammatory process results in connective tissue breakdown as the result of an imbalance of MMPs over the tissue inhibitors of MMPs (TIMPs)^35^. Here, the inflammatory *in vitro* model decreased collagen III α1 (COL3A1) mRNA expression and increased MMP1/TIMP1 mRNA ratio, mimicking the molecular events observed in periodontal disease (Fig. 1C). Quercitrin treatment downregulated MMP1 IL-1β-induced expression at day 1 and 14, and upregulated TIMP1 IL-1β-decreased expression at day 3 and 14, resulting in a decreased MMP1/TIMP1 mRNA ratio at short- and long-term (data not shown), in line with previous results without IL-1β stimulation^13^. Also, COL3A1 expression increased in all quercitrin-treated groups. Furthermore, quercitrin prevented the decrease in TIMP1 and COL3A1 after IL-1β stimulation (preventive approach). Therefore, quercitrin could contribute to the regeneration of functional connective tissue destroyed by inflammation.

### Quercitrin increases osteogenic differentiation of hMSCs

**Figure 3:**
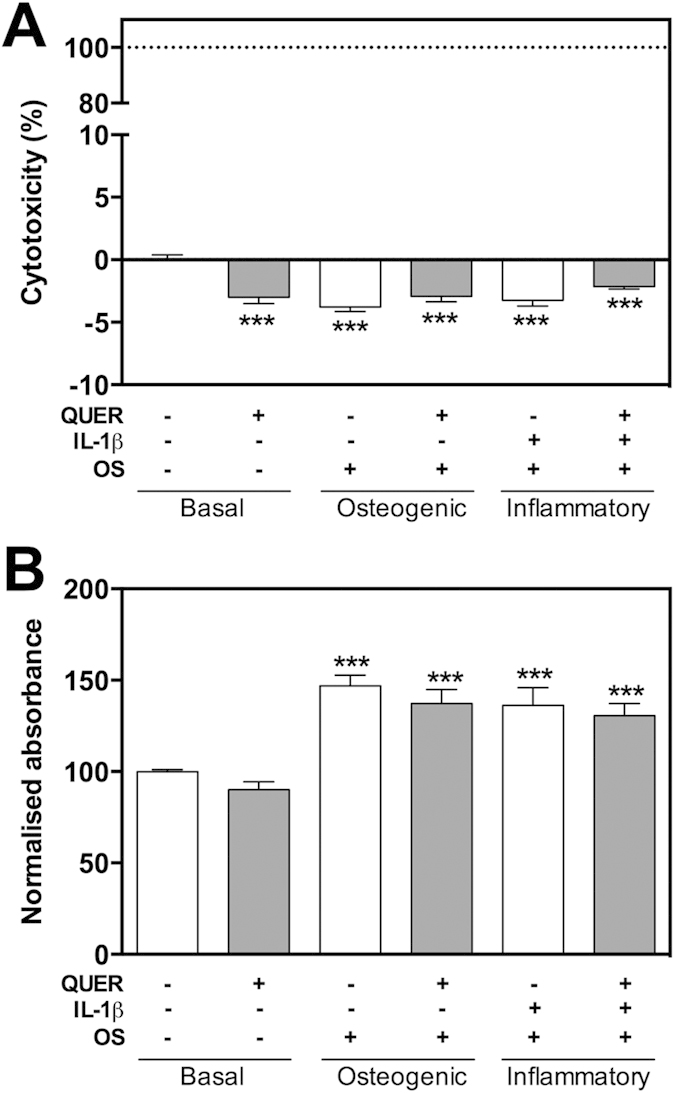
Quercitrin did not show cytotoxic effects on hMSCs. Cells were grown under six different conditions: basal media; basal media with 200 μM quercitrin (QUER); osteogenic media (OS); osteogenic media with 200 μM quercitrin; osteogenic media supplemented with interleukin-1 beta (IL-1β); and osteogenic media supplemented with IL-1β and quercitrin. **(A)** Cytotoxicity measured after 2 days. Dotted line represents high control (100% cytotoxicity). **(B)** Metabolic activity measured after 19 days. Values represent the mean ± SEM of two independent experiments. One, two and three symbols represent a significant difference between two groups with P ≤ 0.05, P < 0.01 and P < 0.001, respectively: (*) treatment *versus* control (vehicle).

**Figure 4:**
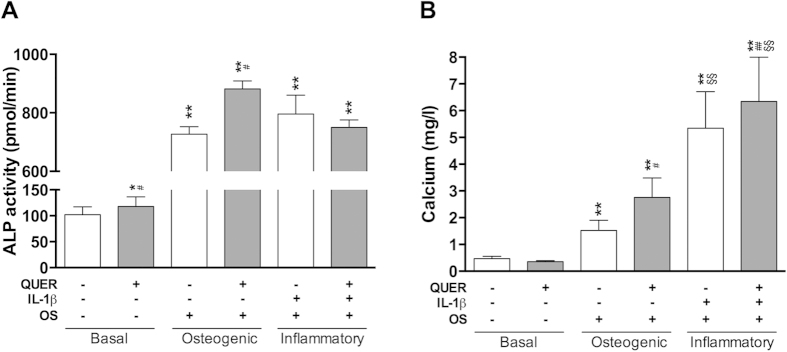
Quercitrin increased the osteoblastic differentiation of hMSCs. (**A**) ALP activity and (**B**) calcium content determined after 19 days of hMSCs growth under six different conditions: basal media; basal media with 200 μM quercitrin (QUER); osteogenic media (OS); osteogenic media with 200 μM quercitrin; osteogenic media supplemented with interleukin-1 beta (IL-1β); and osteogenic media supplemented with IL-1β and quercitrin. Values represent the mean ± SEM of two independent experiments. One, two and three symbols represent a significant difference between two groups with P ≤ 0.05, P < 0.01 and P < 0.001, respectively: (*) treatment versus control (vehicle); (#) quercitrin versus vehicle; (§) inflammation groups *versus* osteogenic groups.

Bone-marrow MSCs were used in this study since they have the capacity to promote the regeneration of alveolar bone, cementum and periodontal ligament^36^,^37^ and that bone-marrow MSCs are comparable to periodontal ligament MSCs in their differentiation capacity and ability to regenerate periodontal bone^38^,^39^. This study proves that quercitrin does not show cytotoxic effects (Fig. 3) and significantly increases ALP activity in basal and osteogenic media, and mineralization in osteogenic and inflammatory situations (Fig. 4) in hMSCs. No significant differences were observed in calcium content of cells treated with quercitrin in basal media compared to control. Furthermore, under IL-1β stimulation, final calcium content increased while ALP activity decreased (Fig. 4). During osteoblastic differentiation, ALP peaks at the maturation phase and decreases at the mineralization phase. Therefore, in basal conditions data suggests that cells are in early differentiation phases while in the presence of IL-1β and osteogenic media the decreased ALP activity together with the increased mineralization point to a shifted hMSCs differentiation to earlier time points, in agreement with previous reports^40^,^41^. This might be explained by the increased metabolic activity in the presence of osteogenic media (Fig. 3B). However, quercitrin addition to the IL-1β-stimulated osteogenic group increased the final calcium content almost 20%, while quercitrin addition to the osteogenic group increased it 80%, although calcium accumulation was higher in the inflammatory groups. It has been demonstrated that IL-1β and dexamethasone increase osteoblast mineralization through two different mechanisms, while dexamethasone increases ALP activity^40^,^41^, IL-1β decreases inhibitory pyrophosphate anions, an atypical biochemical mechanism without differentiating into osteoblasts^42^. The results presented here show that quercitrin increases ALP activity and mineralization of hMSCs, indicating that quercitrin enhanced the osteoblastic differentiation of hMSCs even in the presence of IL-1β, which is supported by a previous study with mouse pre-osteoblasts^14^.

Current research in periodontal regeneration is focused in discovering new bioactive molecules, improving stem cell implantation and developing biomaterials that act as scaffolds or as drug delivery systems^43^. Quercitrin might have different applications in periodontal regeneration since it is a bioactive molecule with antibacterial, anti-oxidant and anti-inflammatory properties and it promotes soft and hard tissue regeneration^13^,^14^,^28^,^29^. Besides, quercitrin could decrease the antibiotic administration and increase the safety of anti-inflammatory drugs currently used for periodontal disease treatment^3^. Regarding to bone regeneration, stem cell-based periodontal regeneration is a promising therapeutic option^37^. Furthermore, the inhibition of osteoblastic differentiation of bone progenitor cells from the periodontal tissue has been suggested to be dependent on the local environment^43^,^44^. Here, quercitrin promoted the osteoblastic differentiation of hMSCs even in an inflammatory situation; therefore quercitrin could help hard tissue to regenerate itself. Furthermore, previous results revealed that quercitrin decrease osteoclast formation in RAW264.7 cells^14^, together with the present results, quercitrin might show an anabolic effect on bone formation. Regarding the development of new biomaterials, our group has also used quercitrin to functionalize Ti-surfaces, showing promising results^45^. Therefore, it is hypothesized that quercitrin could be used in other applications to improve periodontal regeneration. For instance, antioxidants added to oral hygiene products improve periodontal disease indexes^46^,^47^. Thus, we propose the use of quercitrin as a pharmacological agent in the form of toothpastes, mouth rinses or suitable formulations (e.g. hydrogels) for topical application. Future studies should confirm the effects of quercitrin *in vivo*.

In conclusion, the positive effects of quercitrin on cells from soft and hard tissue under inflammatory conditions are suggested using human primary cultures of gingival fibroblasts and mesenchymal stem cells. Quercitrin decreases the release of the inflammatory mediator PGE2 and partially re-establishes the collagenolytic metabolism induced by IL-1β stimulation on primary human gingival fibroblasts. Furthermore, quercitrin increases ALP activity and mineralization, thus, enhancing human mesenchymal stem cells differentiation towards the osteoblastic lineage. These findings suggest quercitrin as a bioactive molecule that could create a microenvironment suitable for soft and hard tissue regeneration and therefore enhance periodontal regeneration.

## Additional Information

**How to cite this article**: Gómez-Florit, M. *et al.* Quercitrin for periodontal regeneration: effects on human gingival fibroblasts and mesenchymal stem cells. *Sci. Rep.* **5**, 16593; doi: 10.1038/srep16593 (2015).

## Figures and Tables

**Table 1:** Sense (S) and antisense (A) primers used in the real-time PCR of reference and target genes.

Gene (Gene symbol)	Primer sequence (5′- 3′)	Product size(bp)
Beta-actin (ACTBL2)	S: CTGGAACGGTGAAGGTGACA	136
	A: AAGGGACTTCCTGTAACAATGCA	
Collagen III α1 (COL3A1)	S: GGCCTACTGGGCCTGGTGGT	190
	A:CCACGTTCACCAGGGGCACC	
Cyclooxygenase-2 (COX2)	S: ATGGGGTGATGAGCAGTTGT	221
	A: GAAAGGTGTCAGGCAGAAGG	
Glyceraldehyde 3-phosphate dehydrogenase (GAPDH)	S: TGCACCACCAACTGCTTAGC	87
	A: GGCATGGACTGTGGTCATGAG	
Interleukin-6 (IL6)	S: AGGAGACTTGCCTGGTGAAA	196
	A: GCATTTGTGGTTGGGTCAG	
Interleukin-8 (IL8)	S: GGTGCAGTTTTGCCAAGGAG	183
	A: TTCCTTGGGGTCCAGACAGA	
Matrix metalloproteinase-1 (MMP1)	S: TGTCAGGGGAGATCATCGGGACA	177
	A: TGGCCGAGTTCATGAGCTGCA	
Metalloproteinase inhibitor-1 (TIMP1)	S: TTCCGACCTCGTCATCAGGG	144
	A: TAGACGAACCGGATGTCAGC	

**Table 2:** Effect of IL-1β stimulation on hGF. Data were normalized to reference genes, expressed as percentage of control (no stimulation) which was set to 100%.

IL-1β (ng/ml)	mRNAlevels (% of control)		
	IL6	IL8	MMP1
0	100 ± 8	100 ± 9	100 ± 4
0.1	3952 ± 509***	81456 ± 12260***	333 ± 27***
1	3832 ± 477***	80414 ± 11765***	369 ± 37***
10	3709 ± 410***	76026 ± 11161***	362 ± 36***

Values represent the mean ± SEM of two independent experiments. Differences between groups: ***P < 0.001 treatment *versus* control.
